# The Role of Virtual and Augmented Reality in Transsphenoidal Surgical Approaches to the Sellar and Parasellar Area—A Systematic Review

**DOI:** 10.3390/jcm15114142

**Published:** 2026-05-27

**Authors:** Kristian Bechev, Daniel Markov, Vladimir Aleksiev, Galabin Markov, Elena Poryazova, Antoaneta Fasova

**Affiliations:** 1 Department of Anatomy, Histology and Cytology, Faculty of Medicine, Medical University of Plovdiv, 4002 Plovdiv, Bulgaria; antoaneta.fasova@mu-plovdiv.bg; 2 Neurological Surgery, Pulmed University Hospital, 4000 Plovdiv, Bulgaria; 3 Department of General and Clinical Pathology, Medical University of Plovdiv, 4002 Plovdiv, Bulgaria; daniel.markov@mu-plovdiv.bg (D.M.); eporiazova@abv.bg (E.P.); 4 Department of Clinical Pathology, UMHAT “Pulmed”, 4002 Plovdiv, Bulgaria; 5 Department of Thoracic Surgery, UMHAT “Kaspela”, 4002 Plovdiv, Bulgaria; vl_alex@abv.bg; 6 Department of Cardiovascular Surgery, Medical University of Plovdiv, 4002 Plovdiv, Bulgaria; 7 Faculty of Medicine, Medical University of Plovdiv, 4002 Plovdiv, Bulgaria; gabi_markov@abv.bg; 8 Clinical and Experimental Morphology Division, Research Institute, Medical University of Plovdiv, 4000 Plovdiv, Bulgaria

**Keywords:** virtual reality, augmented reality, transsphenoidal surgery, pituitary, skull base, surgical navigation, virtual simulation

## Abstract

**Background/Objectives**: Transsphenoidal surgery has become the gold standard for the treatment of sellar and parasellar lesions, but it remains associated with significant anatomical challenges and the risk of intraoperative complications. The limitations of conventional imaging in depicting the complex three-dimensional anatomy of the skull base have led to a growing interest in virtual (VR) and augmented reality (AR) technologies, which offer enhanced spatial visualization, preoperative simulation, and image-guided intraoperative navigation. This systematic review aims to evaluate the current evidence on the role of virtual and augmented reality in transsphenoidal surgical interventions, with a focus on their impact on preoperative planning, intraoperative orientation, surgical outcomes, and neurosurgical training. **Methods**: A systematic literature search was conducted in accordance with PRISMA 2020 guidelines across PubMed, Scopus, and Web of Science for the period 2015–2025. MeSH terms and free-text keywords related to transsphenoidal surgery, sphenoid sinus anatomy, and VR/AR technologies were combined using Boolean operators. Risk of bias was assessed using RoB 2.0 for RCTs; methodological quality was assessed using the Newcastle–Ottawa Scale for observational studies and AMSTAR 2 for systematic reviews. Clinical, morphometric, and experimental studies evaluating VR/AR applications were included. Data were extracted using a standardized protocol and synthesized through qualitative analysis, with subgroup analysis by technology type (VR vs. AR) and clinical application domain. **Results**: A total of 218 publications were identified, of which 52 met the inclusion criteria (clinical studies n = 12, simulation and technology studies n = 30, morphological studies n = 10). VR-based three-dimensional reconstructions were consistently associated with improved preoperative spatial orientation and anatomical landmark recognition. AR systems demonstrated a meaningful contribution to intraoperative navigation, with reported reductions in time to target and improved visualization of critical neurovascular structures. VR platforms showed high effectiveness in surgical training, with shorter learning curves and improved technical performance. However, the majority of included studies were small observational cohorts, simulation studies, or expert overviews, with substantial heterogeneity in methodology, technology platforms, and outcome measures, precluding quantitative meta-analysis. **Conclusions**: Virtual and augmented reality represent clinically promising adjuncts to transsphenoidal surgery, with demonstrated benefits in preoperative planning, intraoperative navigation, and surgical training. These conclusions should be interpreted in the context of a predominantly early-phase and heterogeneous evidence base. Standardized protocols, larger prospective studies, and randomized trials are needed before the integration of VR/AR with navigation systems and artificial intelligence can be established as a routine component of personalized transsphenoidal surgery.

## 1. Introduction

Transsphenoidal surgery is a leading and widely used approach for the surgical treatment of pituitary and parasellar tumors, gradually establishing itself as the “gold standard” due to its minimally invasive nature, lower postoperative morbidity, and faster patient recovery compared to classical craniotomy approaches. However, interventions through the sphenoid sinus are associated with a number of technical and anatomical challenges resulting from the significant variability in the morphology of the sinus, as well as its close proximity to essential vascular–neural structures [1,2,3,4,5,6].

The sphenoid sinus (sinus sphenoidalis) is located in the central region of the skull base and exhibits an extremely rich morphological diversity, varying both in terms of the degree of pneumatization and the number and direction of the intersinus septa. The presence of asymmetric cavities, variations in the thickness of the lateral and superior walls, and septa that are fixed directly to the internal carotid artery or optic nerve represent significant risk factors for intraoperative complications [7,8,9,10,11]. Surgical safety and effectiveness depend largely on the early recognition of these variations and on detailed preoperative planning. Classical imaging methods—computed tomography (CT) and magnetic resonance imaging (MRI)—remain the main diagnostic tools, providing valuable information about the anatomical relationships. CT in particular is the “gold standard” for assessing the bone morphology and the degree of pneumatization of the sphenoid sinus, while MRI is an indispensable method for visualizing soft tissue structures and neoplasia in the pituitary and parahypophyseal region [6,12,13,14,15,16,17,18].

The traditional two-dimensional representation of complex three-dimensional anatomy often proves to be insufficient, especially in patients with atypical anatomical features. The lack of spatial perspective can increase the risk of intraoperative disorientation and complications. In this context, in the last decade, virtual reality (VR) and augmented reality (AR) have established themselves as innovative technologies offering new possibilities for surgical planning, intraoperative navigation and training of neurosurgical residents [5,15,16,19,20]. VR allows for the reconstruction of individualized anatomical models created from high-resolution DICOM CT data, which can be explored in an interactive three-dimensional environment. This creates the opportunity to “virtually enter” the sphenoid sinus and preoperatively rehearse the surgical approach, which significantly improves spatial orientation and awareness of risk areas [5,11,16,17,20,21,22]. Augmented reality, in turn, provides a layering of virtual images on the real surgical field, aiding intraoperative navigation and reducing the likelihood of damage to underlying anatomical structures. The combination of VR and AR with traditional imaging methods offers a new model for personalized surgery, in which the individual anatomy of the patient becomes a dynamic simulation scenario. In addition to preoperative preparation, these technologies are also used in medical education, providing realistic training conditions for residents and young surgeons without exposing patients to risk [13,17,22,23,24].

Given these perspectives, the present review aims to summarize current data on the application of VR and AR technologies in transsphenoidal surgical interventions to the pituitary gland and parasellar space. In this way, not only the benefits of integrating these technologies but also the challenges and prospects for their future development will be outlined.

## 2. Materials and Methods

This systematic review was conducted in accordance with the updated PRISMA 2020 Main Checklist (Preferred Reporting Items for Systematic Reviews and Meta-Analyses), which provide a standardized framework for the systematic identification, selection, and synthesis of the scientific literature. The methodological design was pre-structured based on the PICO model in order to formulate clearly defined and clinically relevant research questions, as well as minimize the risk of systematic errors. The PICO framework includes the following indicators:

❖ Population (P): patients undergoing transsphenoidal pituitary surgery;
❖ Intervention (I): application of virtual or augmented reality (VR/AR), based on imaging diagnostics (CT/MRI);
❖ Comparison (C): standard methods for preoperative planning without VR/AR;
❖ Outcome (O): improved spatial orientation, reduced surgical complications, shortened operative time, and increased educational effect.

The research question was aimed at assessing the role of virtual and augmented reality (VR/AR), based on high-resolution imaging data, in the optimization of transsphenoidal surgical approaches to the sellar and parasellar regions. Specifically, the analysis focused on the potential of these technologies to improve spatial orientation, reduce the incidence of intraoperative complications, shorten operative time, and optimize the training process in neurosurgical interventions.

A systematic literature search was conducted in the electronic databases PubMed/MEDLINE, Scopus, and Web of Science, covering the period from January 2015 to December 2025. The final search was executed in December 2025. A structured search strategy was applied independently in each database, combining controlled vocabulary (MeSH terms for PubMed) with free-text keywords using Boolean operators (AND, OR). The search was organized around three conceptual domains: (1) the surgical approach—transsphenoidal, endonasal, pituitary, sellar, parasellar, skull base; (2) the technology—virtual reality, augmented reality, extended reality, mixed reality, surgical simulation; and (3) the anatomical target—sphenoid sinus, sella turcica. The complete search strings applied in each database are provided in Table 1. A supplementary manual search of the reference lists of all included publications was performed to identify additional relevant studies not captured by the database search.

The primary search was restricted to publications from 2015 onwards, reflecting the period during which VR/AR technologies became sufficiently mature for surgical application. Three studies published prior to this window (Kawamata et al. 2002 [25]; Rosseau et al. 2013 [26]; Cho et al. 2010 [27]) were retained in the final analysis as historically significant references that established the foundational principles underlying current VR/AR applications in transsphenoidal surgery; these studies are clearly identified in Table 1 and are not counted toward the primary evidence synthesis.

To ensure methodological efficiency, clear inclusion and exclusion criteria were defined. Studies that examined the morphology of the sphenoid sinus using modern imaging methods, as well as studies evaluating the application of VR and/or AR technologies in the context of transsphenoidal surgery, were included in the analysis. Clinical (prospective and retrospective), morphometric and experimental studies were eligible, provided that they contained a clearly described methodology and provided quantitative or qualitative results, according to the objectives of the analysis. Individual case studies, publications without direct anatomical or clinical correlation, articles without access to full text, and those outside the defined time range or in a language other than English were excluded from the study.

The selection of studies was carried out through a two-stage process by two independent researchers, which aims to minimize selection bias. Initially, titles and abstracts were screened to eliminate publications outside the scope of the given study, followed by a full-text analysis of potentially relevant articles. In the event of disagreement between the two researchers, decisions were made by consensus, and if necessary, a third expert in the field was involved. Cohen’s coefficient (Cohen’s kappa) was used to quantitatively assess observer agreement, which contributes to increasing the reliability and reproducibility of the selection process.

Data extraction was performed using a standardized form developed specifically for this study to ensure a uniform approach to the analysis of included publications. For each study, detailed data were collected on authors, year of publication, geographical origin, study design, sample size, and patient demographics. Special emphasis was placed on the characteristics of the VR/AR technologies used, including platform type, visualization method, and their specific application—preoperative planning, intraoperative navigation, or training. The main endpoints included complication rate, operative time, degree of surgical orientation, and training effectiveness.

Given the heterogeneity of the included studies, an additional stratified analysis (subgroup analysis) was performed, which allowed wanted to investigate the relationships between different types of studies and outcomes in more depth. The studies were grouped into two main subgroups: (1) studies evaluating VR/AR technologies in a simulation environment and (2) clinical studies analyzing real surgical outcomes. In addition, a subgroup assessment was performed according to the type of technology used (VR vs. AR) as well as according to the clinical context (preoperative planning vs. intraoperative navigation). This approach allowed for more precise interpretation of the data and identification of potential differences in the effectiveness of different technological solutions.

The methodological quality and risk of bias of the included studies were assessed using validated instruments selected according to study design. The Cochrane Risk of Bias Tool 2.0 (RoB 2.0) was applied to randomized controlled trials, the Newcastle–Ottawa Scale (NOS) was used for observational cohort and case–control studies, and AMSTAR 2 was applied to included systematic reviews. Quality appraisal results were used to contextualize the interpretation of findings and are reported narratively in the Discussion. Given the predominantly exploratory and heterogeneous character of the included literature—spanning clinical cohorts, simulation studies, technical feasibility reports, and secondary analyses—formal methodological quality appraisal and, where applicable, risk-of-bias assessment was employed to qualify the level of confidence attributable to individual study findings rather than as a basis for exclusion. The overall methodological quality of the evidence base, and the associated risk of bias where formally assessed, was judged to be predominantly low to moderate by these criteria, reflecting the early developmental stage of VR/AR technology in this surgical context.

Due to the substantial heterogeneity between the included studies in terms of design, patient populations, technology platforms, and outcome measures, quantitative meta-analysis was not feasible. Data were therefore synthesized through qualitative narrative analysis. Included systematic reviews and meta-analyses were treated as secondary evidence and used to contextualize primary findings, rather than as equivalent primary data sources.

For better interpretation of the heterogeneous data included in the study, they were systematized and categorized in Table 2 and Table 3 according to their design, technology type and clinical application.

### AI Statement

No artificial intelligence was used in the manuscript, except for translating and improving the linguistic and grammatical aspects of the English text. Deepl Translate was used as a tool for this purpose.

## 3. Results

**Figure 1:**
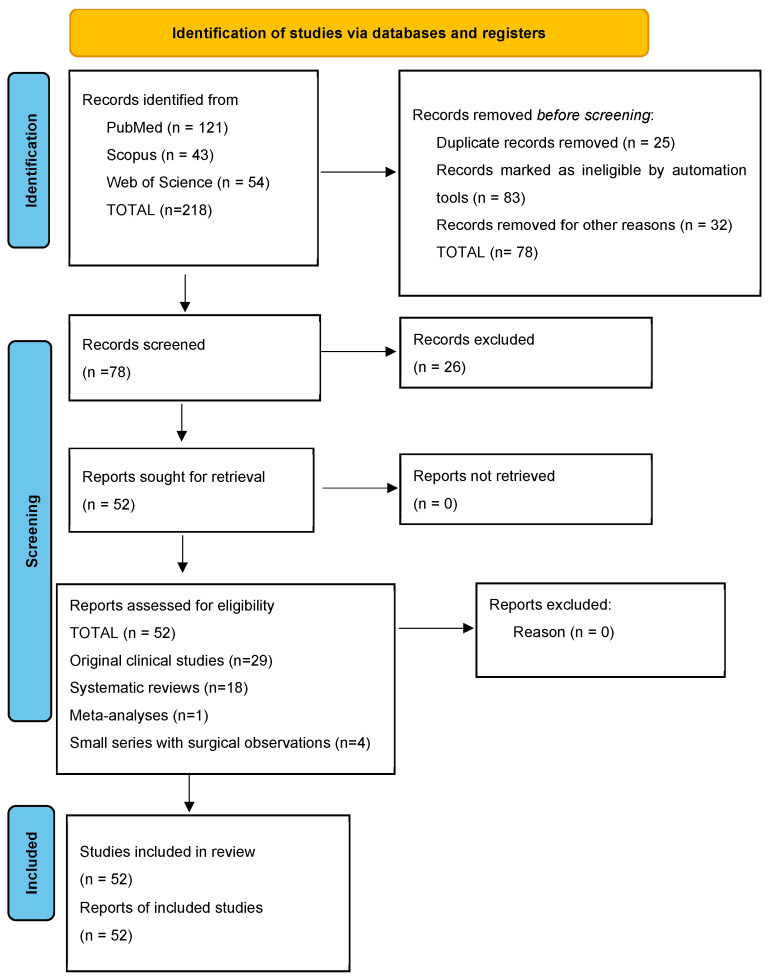
PRISMA diagram of the scientific materials included in the systematic review [54].

The initial systematic search identified a total of 218 publications from the PubMed, Scopus, and Web of Science databases. After removing duplicate materials, 193 records were screened, of which 115 were excluded after reviewing the titles and abstracts due to the lack of a direct connection to the topic (they were systematized and evaluated by two independent experts from the author team). A full-text analysis was performed on 78 publications, 25 of which were excluded due to non-compliance with the inclusion criteria (lack of VR/AR application, insufficient methodological clarity, or different clinical focus from the assigned topic). A total of 52 studies presented in the PRISMA diagram were included in the final analysis (Figure 1).

The analysis of the included publications showed a pronounced heterogeneity in terms of design, population and technologies used. For better interpretation, the studies were classified into three main groups, with the main data extracted from Table 1 presented in the Section 2:

❖ VR/AR technological and simulation studies (n = 30);
❖ Morphological and anatomical studies (n = 10);
❖ Clinical and surgical studies (n = 12).

This subdivision of the included studies allowed a more precise analysis of the role of virtual and augmented reality in the different stages of transsphenoidal surgery.

### 3.1. Effects of Virtual Reality (VR)

Virtual reality has demonstrated significant application in preoperative planning and training of young neurosurgeons (this group includes neurosurgeons with less than 5 years of experience in the specialty). The included studies show that VR-based three-dimensional reconstructions, built on the basis of CT and MRI data, allow detailed visualization of the individual anatomy of the patient, and the surgical approach can be simulated in a VR environment. This leads to a significant improvement in spatial orientation and recognition of anatomical landmarks. VR platforms demonstrate high efficiency in training residents and young neurosurgeons, by providing a realistic simulation environment and the ability to repeatedly perform surgical accesses to target areas. Several studies report a significant shortening of the learning curve and improvement in technical skills, including higher accuracy of movements and a reduction in intraoperative errors.

### 3.2. Effects of Augmented Reality (AR)

Augmented reality is emerging as a key technology for intraoperative navigation. Clinical studies have shown that AR systems based on overlaying virtual anatomical structures on a real surgical field significantly improve orientation during the surgical procedure.

Reported results include:

❖ Reduction in time to reach the sellar region;
❖ More precise identification of critical anatomical structures such as the internal carotid artery and optic nerve;
❖ Reduction in unintended instrument movements, improvement in surgical precision;
❖ Some studies have reported a reduction in operative time by approximately 20–25%, which highlights the clinical relevance of AR technology.

### 3.3. Subgroup Analysis (VR vs. AR)

The subgroup analysis (summarized in Table 2, presented in the Section 2) demonstrates a clear functional distinction between the two technologies. Virtual reality has a dominant role in preoperative preparation and education of interested groups, while augmented reality is a leading tool in intraoperative navigation. Their combined application creates a synergistic effect, in which VR supports the planning and understanding of anatomical structures and the relevant variations, and AR provides real-time visualization and surgical control during surgical manipulation.

### 3.4. Morphological Factors and Clinical Significance

Morphological studies confirm the significant variability of the sphenoid sinus, including differences in the degree of pneumatization, the configuration of the intersinus septa, and the relationships with adjacent neurovascular anatomical structures. These features have a direct impact on surgical risk and the complexity of the surgical activity. The integration of VR/AR technologies with morphometric data allows for better identification of risk areas and optimization of the surgical strategy, especially in patients with atypical variations.

### 3.5. Limitations of the Available Data

Despite the reported positive effects, the analysis shows that the available studies are characterized by:

❖ Limited samples;
❖ Heterogeneity in methodology;
❖ Lack of standardized protocols;
❖ Insufficient number of randomized controlled trials.

These factors limit the possibility of quantitative meta-analysis and require caution in interpreting the results.

## 4. Discussion

This systematic review synthesizes data from 52 studies examining the application of virtual and augmented reality in transsphenoidal surgery. The available evidence indicates that both technologies offer clinically meaningful contributions to spatial orientation, surgical precision, and neurosurgical training, with a functionally distinct role for each modality. These findings should, however, be contextualized within the overall quality and heterogeneity of the included evidence base.

### 4.1. Study Heterogeneity and Evidence Hierarchy

A fundamental methodological consideration in interpreting the findings of this review is the pronounced heterogeneity of the included literature. The 52 included studies represent at least five distinct categories: clinical outcome studies (n = 12), VR/AR simulation and technology studies (n = 30), morphometric anatomical analyses (n = 10), as well as several systematic reviews and meta-analyses that were incorporated as secondary evidence to provide contextual support rather than as equivalent primary sources. Outcome measures across these categories—ranging from operative time and extent of resection in clinical studies, to validity scores and task error rates in simulation studies, and morphometric parameters in anatomical analyses—are not directly comparable and cannot be pooled quantitatively [54].

Applying the Oxford Centre for Evidence-Based Medicine hierarchy, the evidence base for VR/AR in transsphenoidal surgery is predominantly at levels 3 to 5. The single meta-analysis identified (Sung et al. 2024) evaluated VR in healthcare education broadly and was not specific to transsphenoidal surgery [39]. No randomized controlled trials with adequate statistical power focusing on this specific surgical context were identified. The majority of clinical studies were small retrospective cohorts (Goto et al., n = 15; Tortolero et al., n = 18; Novák et al., n = 6; Zhang et al., n = 5; Carl et al., n = 47; Bopp et al., n = 165), and simulation studies involved small numbers of participants evaluated under controlled non-clinical conditions [12,15,36,40,45,50]. These characteristics inherently limit the generalizability of conclusions and underscore the need for caution in extrapolating findings to routine clinical practice. Future systematic reviews on this topic would benefit from formally separating simulation-based training evidence from clinical outcome evidence, as the applicable quality assessment frameworks and interpretive standards differ substantially between these categories.

### 4.2. Anatomical Context and Implications for VR/AR Integration

The morphological variability of the sphenoid sinus including differences in pneumatization type, intersinus septa configuration, and proximity to the internal carotid artery and optic nerve is a well-established driver of surgical risk in transsphenoidal approaches [7,8,9,27,47]. Morphometric studies included in this review confirm that these variations are clinically significant and often inadequately captured by standard two-dimensional imaging alone [11,29,35,44]. This anatomical complexity provides the principal rationale for integrating VR/AR technologies, which offer individualized three-dimensional spatial modelling that extends beyond what conventional CT and MRI can provide in a standard planar format [18,32,49].

### 4.3. Virtual Reality: Preoperative Planning and Surgical Training

Virtual reality is consistently associated with improvements in preoperative spatial orientation and anatomical landmark recognition across the included studies. VR-based three-dimensional reconstructions derived from CT and MRI data allow individualized simulation of the surgical approach, enabling the surgeon to rehearse anatomical navigation before entering the operating room. Lee et al. 2025 and Wu et al. 2015 demonstrated improvements in spatial visualization through three-dimensional reconstruction, while Inoue et al. 2015 highlighted the utility of 3D imaging for anatomical orientation in endonasal approaches [6,28,30]. Munawar et al. 2024 and Filimonov et al. 2022 further demonstrated the feasibility of patient-specific VR modelling with direct implications for personalized surgical planning [3,23,41,43]. The methodological value of CT-based three-dimensional VR simulation for procedural planning in anatomically constrained regions has been further demonstrated in adjacent anatomical corridors: Pušnik et al. 2026 applied CTA-derived VR simulation to compare the feasibility and vascular safety of suprazygomatic and infrazygomatic maxillary nerve block approaches in 89 children, confirming that immersive spatial modelling can reliably identify optimal trajectories and quantify safety margins a principle directly applicable to the complex anatomy of the sellar and parasellar region [53].

In the context of surgical training, VR simulators have shown notable effectiveness in shortening the learning curve and improving technical performance under controlled conditions. Rosseau et al. 2013, Santona et al. 2023, and Shao et al. 2020 reported improvements in technical skills and procedural accuracy following VR-based training, with the latter demonstrating superiority over traditional teaching methods in a randomized design (n = 30) [13,14,26]. The meta-analysis by Sung et al. 2024, encompassing 45 randomized controlled trials in healthcare education, broadly confirmed the effectiveness of VR as a training modality, though its findings are not specific to transsphenoidal surgery and should be applied cautiously in this context [19,39].

### 4.4. Augmented Reality: Intraoperative Navigation

Augmented reality has demonstrated a more direct clinical role in the intraoperative phase. Systems that overlay virtual anatomical structures including the internal carotid artery, optic nerve, and tumour margins onto the real endoscopic field have been associated with improved intraoperative orientation and reduced cognitive load from screen-switching [25]. Carl et al. 2019 reported that microscope-based AR was reliably integrated into the surgical workflow in 47 of 288 consecutive transsphenoidal procedures, with smooth registration using intraoperative CT [36]. Goto et al. 2023 evaluated their AR navigation system in 15 consecutive patients, reporting a mean efficacy score of 4.7 out of 5 and noting potential reductions in operative time [40]. Bopp et al. 2022 described AR integration in a series of 165 patients, with AR support associated with improved intraoperative orientation without adverse effects on workflow [12]. Zhang et al. 2025 achieved a target registration error of 2.23 ± 0.57 mm in five patients, meeting established precision standards for neuronavigation [50].

Systematic reviews by Meola et al. 2017, Campisi et al. 2023, and Begagić et al. 2024 broadly confirm that AR technologies appear to improve surgical accuracy and safety, while consistently highlighting the absence of standardization and the variability of results across platforms and institutions [16,35,37]. The recent systematic review by Thavarajasingam et al. 2022 noted that AR outcomes were broadly comparable to conventional neuronavigation in the studies reviewed, suggesting that AR may function as a useful adjunct rather than a replacement for existing navigation systems [52].

### 4.5. Clinical Translation, Implementation Barriers, and Comparison with Conventional Neuronavigation

Despite the promising signals in the available literature, several practical barriers currently limit the routine clinical adoption of VR/AR technologies in transsphenoidal surgery. These barriers require explicit acknowledgement and represent important targets for future research.

*Hardware and economic constraints.* The acquisition and maintenance of AR-capable surgical microscopes, head-mounted displays, and real-time tracking systems represent a substantial capital investment. Commercially available AR navigation systems, such as those integrated into modern operating microscopes (e.g., Zeiss KINEVO with head-up display, Brainlab Curve), require specialized technical support and institutional infrastructure that may not be universally available, particularly in lower-resource settings. VR simulation platforms, while generally less expensive than intraoperative AR systems, still require dedicated hardware and software licensing, as well as trained personnel to manage content updates and equipment maintenance [5,22,45].

*Registration accuracy and intraoperative reliability.* A fundamental challenge for AR neuronavigation is maintaining accurate registration between virtual overlays and the real surgical field throughout the procedure. Intraoperative brain shift—caused by cerebrospinal fluid egress, tissue manipulation, and tumour removal—progressively reduces the accuracy of registration based on preoperative imaging. In transsphenoidal surgery, deformation of sellar contents and the diaphragma sellae during tumour removal may further compromise overlay precision. Automatic registration using intraoperative CT (as described by Carl et al. and Bopp et al.) addresses some of these concerns, but increases radiation exposure and workflow complexity compared to surface-based or fiducial marker registration [12,36]. The target registration errors reported across included studies (e.g., 2.23 ± 0.57 mm by Zhang et al.) are clinically acceptable, but systematic prospective evaluation of overlay accuracy across the full duration of a surgical procedure remains limited in the current evidence base [48,50].

*Learning curve for technology adoption.* The implementation of VR/AR technologies introduces a technology-specific learning curve that is distinct from the surgical learning curve. Experienced neurosurgeons accustomed to established neuronavigation systems and classical microscopic techniques may encounter an adaptation period associated with the interpretation of overlaid virtual structures, calibration of AR displays, and workflow integration. Several included studies note that surgeons rated AR systems as less useful in specific cases Goto et al. reported that 2 of 15 evaluations scored the AR system as less useful than conventional navigation, citing difficulties in depth perception [40]. This highlights the importance of structured technology-specific training programmes for both trainee and experienced surgeons before clinical deployment [5,31,34].

*Comparison with current neuronavigation systems.* A clinically relevant question that the current evidence does not fully resolve is what AR adds beyond established frameless neuronavigation platforms (e.g., Brainlab Curve, Medtronic Stealth Station) that are already in wide clinical use. Standard neuronavigation provides accurate pointer-based localization of surgical instruments relative to preoperative imaging, and is considered the current standard of care in most neurosurgical centres performing transsphenoidal surgery. AR systems extend this by projecting anatomical structures directly into the surgeon’s operative field of view, potentially reducing the cognitive load of mental image fusion between navigation monitor and endoscopic view. However, the incremental clinical benefit—in terms of complication reduction, extent of resection, or operative time—over high-quality standard neuronavigation has not been demonstrated in adequately powered comparative studies. The systematic review by Thavarajasingam et al. 2022 found outcomes broadly comparable between AR and conventional navigation, and Bopp et al. 2022 similarly found AR to be a complementary rather than transformative addition to their workflow [2,12,52].

*Clinically adopted versus experimental technologies.* It is important to distinguish between VR/AR applications that have reached clinical implementation and those that remain predominantly in the experimental or feasibility phase. Among the included studies, microscope-based AR navigation systems (Carl et al.; Bopp et al.) and AR-enhanced endoscopic platforms (Goto et al.; Tortolero et al.; Zhang et al.) represent the most clinically mature applications, having been tested in live surgical series [12,36,40,45,50]. AI-driven real-time anatomical localization systems such as PitSurgRT (Mao et al.) and automatic endoscopic registration systems (Enkaoua et al.) represent a more early-stage category where clinical validation is still ongoing [1,38,46]. Most VR simulation platforms described in the training literature (Rosseau et al.; Nillahoot et al.; Munawar et al.) have been evaluated in controlled laboratory or educational settings and have not yet been tested for direct transfer of training to improved operative outcomes in clinical practice. Clearly communicating this distinction is essential to avoid overstating the current clinical readiness of these technologies [26,43,51].

### 4.6. Limitations

The present review has several limitations that should be acknowledged. The primary search covered the period 2015–2025; three studies published prior to this window (Rosseau et al. 2013; Cho et al. 2010) were included as historical contextual references given their foundational contributions to the field [26,27]. The included literature is characterized by substantial heterogeneity in study design, technology platforms, patient populations, and outcome measures, which precluded quantitative meta-analysis and limits the strength of comparative conclusions. Simulation-based studies—which represent the largest category of included evidence—demonstrate inherently limited external validity, as performance on a simulator does not automatically translate to improved outcomes in live surgery; this transfer of training effect has not been systematically evaluated in the transsphenoidal context. Many clinical studies had small sample sizes, lacked control groups, and did not apply blinding, reflecting fair to poor methodological quality on the Newcastle–Ottawa Scale and, in the single RCT included, some concerns regarding risk of bias on RoB 2.0. The absence of standardized reporting formats for AR accuracy metrics further complicates cross-study comparison. Finally, psychophysiological limitations—including eye fatigue, spatial disorientation, and workflow disruption—have been noted by some authors and represent an underexplored aspect of VR/AR implementation in clinical practice [5,6,13,20,22,24].

### 4.7. Future Directions

Looking forward, the convergence of VR/AR with artificial intelligence-driven anatomical recognition, robotic assistance, and real-time intraoperative imaging may eventually lead to more sophisticated and integrated surgical platforms. AI-enhanced segmentation of critical structures—as explored by Mao et al. 2024 and Kenig et al. 2024—could improve the accuracy and reliability of AR overlays while reducing the manual preparation time required for current systems [33,46]. The concept of extended reality (XR), reviewed by Sanker et al. 2025, points toward a convergent technological trajectory [42]. However, realizing these possibilities will require standardized outcome reporting, multicentre prospective trials, and health technology assessments that account for the full cost–benefit profile of VR/AR integration across different institutional contexts [4,10].

## 5. Conclusions

Transsphenoidal surgery continues to evolve in response to the anatomical demands imposed by the morphological complexity of the sellar and parasellar region. High-resolution computed tomography remains indispensable for preoperative assessment of sphenoid sinus architecture, but the inherent limitations of two-dimensional imaging in conveying three-dimensional spatial relationships have created a practical rationale for exploring supplementary technologies.

The studies synthesized in this review suggest that virtual reality and augmented reality offer relevant contributions at several stages of the surgical process. VR-based preoperative modelling has been associated with improved spatial orientation and more effective preparation for anatomical challenges, while AR navigation systems have demonstrated feasibility and acceptable registration accuracy in clinical series of varying size. In the domain of surgical education, VR simulators appear to shorten the learning curve for endoscopic endonasal techniques, though the transfer of simulator-acquired skills to real operative performance has not yet been systematically evaluated.

It should be noted, however, that the body of evidence reviewed here consists predominantly of small observational cohorts, technical feasibility reports, and simulation studies. No randomized controlled trials with adequate statistical power were identified, and the heterogeneity of platforms, outcome measures, and patient populations limits the degree to which broad conclusions can be drawn. The absence of standardized protocols and long-term follow-up data means that the precise incremental benefit of VR/AR over established neuronavigation systems—in terms of complication rates, extent of resection, or operative efficiency—remains to be determined through adequately designed prospective research. Taken together, VR and AR technologies represent a clinically promising area of development in transsphenoidal surgery rather than an established standard of care. Their practical integration into routine neurosurgical workflows will depend on advances in registration reliability, reductions in hardware costs, and crucially on the accumulation of higher-quality clinical evidence demonstrating meaningful patient benefit. Whether the convergence of these technologies with artificial intelligence and robotic assistance will substantively change outcomes in this field remains an open question that prospective multicentre studies will need to address.

## Figures and Tables

**Table 1:** Complete database search strings.

Database	Hits (n)	Search String
PubMed/MEDLINE	121	“transsphenoidal” [MeSH Terms] OR “transsphenoidal surgery” [Title/Abstract] OR “endonasal approach” [Title/Abstract] OR “endonasal transsphenoidal” [Title/Abstract] **AND** “virtual reality” [MeSH Terms] OR “augmented reality” [MeSH Terms] OR “virtual reality” [Title/Abstract] OR “augmented reality” [Title/Abstract] OR “extended reality” [Title/Abstract] OR “mixed reality” [Title/Abstract] OR “surgical simulation” [Title/Abstract] OR “surgical simulator” [Title/Abstract] OR “3D reconstruction” [Title/Abstract] **AND** “pituitary” [MeSH Terms] OR “sella turcica” [MeSH Terms] OR “sellar region” [Title/Abstract] OR “parasellar” [Title/Abstract] OR “skull base” [Title/Abstract] OR “sphenoid sinus” [MeSH Terms] OR “sphenoid sinus” [Title/Abstract] OR “pituitary surgery” [Title/Abstract] AND “1 January 2015” [Date—Publication]: “31 December 2025” [Date—Publication]
Scopus	43	TITLE-ABS-KEY “transsphenoidal” OR “endonasal approach” OR “transsphenoidal surgery” OR “endonasal transsphenoidal” **AND** “virtual reality” OR “augmented reality” OR “extended reality” OR “mixed reality” OR “surgical simulation” OR “surgical simulator” OR “3D reconstruction” **AND** (“pituitary” OR “sellar” OR “parasellar” OR “skull base” OR “sphenoid sinus” OR “pituitary surgery” AND PUBYEAR > 2014 AND PUBYEAR < 2026
Web of Science	54	TS = “transsphenoidal” OR “endonasal approach” OR “transsphenoidal surgery” OR “endonasal transsphenoidal” **AND** “virtual reality” OR “augmented reality” OR “extended reality” OR “mixed reality” OR “surgical simulation” OR “surgical simulator” OR “3D reconstruction” **AND** “pituitary” OR “sellar” OR “parasellar” OR “skull base” OR “sphenoid sinus” OR “pituitary surgery” AND PY = (2015–2025)

MeSH = Medical Subject Headings; TS = Topic Search field (Web of Science); TITLE-ABS-KEY = Title, Abstract and Keywords field (Scopus). Search date: December 2025.

**Table 2:** Summary of included studies by design, technology type, clinical application, sample size, level of evidence (LoE), and primary outcome metrics.

No.	Author (Year)	Study Design	Technology	Clinical Application	N	LoE	Primary Outcome Metric	Key Quantitative Result/Main Finding
1	Lee et al. (2025) [28]	Clinical	VR/3D	Preoperative planning	−	4	3D landmark visualization accuracy	Improved spatial orientation (qualitative)
2	Jaworek-Troć et al. (2022) [29]	Morphometric	CT	Anatomy	−	4	Sphenoid ostium dimensions (mm)	Clinically significant anatomical variants identified
3	Muslu et al. (2025) [8]	Morphometric	CT	Anatomy	−	4	Foramen lacerum dimensions (mm)	Population-level morphometric differences documented
4	Wu et al. (2015) [30]	Technical note	3D CT	Preoperative planning	−	4/5	Intracranial landmark localization	Accurate sellar localization confirmed
5	Skvortsova et al. (2025) [31]	Cross-sectional	VR	Training	−	4	VR acceptability score (Likert)	High acceptability among health educators
6	Bue et al. (2024) [7]	Retrospective clinical	CT	Anatomy	−	3	Sinus morphology (CT parameters)	Significant differences between PiTNET subtypes
7	Park & Hwang (2021) [32]	Narrative overview	CT	Anatomy	−	5	N/A (descriptive)	Pneumatization patterns described
8	Kenig et al. (2024) [33]	Systematic review	AI	General	studies	1a	AI validation metrics (accuracy, sensitivity)	AI validated in surgical applications
9	Higa et al. (2025) [34]	Experimental	AR	Intraoperative	N = 11 (2 + 4 + 3 + 4 participants across phases)	4	Design factor ratings (qualitative)	AR design factors evaluated by neurosurgeons
10	Meola et al. (2017) [35]	Systematic review	AR	Navigation	studies	1a	AR navigation accuracy (qualitative)	AR improves accuracy; evidence heterogeneous
11	Carl et al. (2019) [36]	Clinical	AR	Intraoperative	N = 47 patients (from series of 288)	3	Landmark ID accuracy; OR time	AR reliable tool for complicated TSS
12	Begagić et al. (2024) [37]	Systematic review	AR	Navigation	19 studies	1a	Navigation accuracy; complication rate	AR improves visualization and orientation
13	Enkaoua et al. (2025) [38]	Technological	AR	Intraoperative	−	5	Registration accuracy (mm)	Automatic AR registration feasible
14	Ahmadipour et al. (2016) [9]	Clinical	CT	Anatomy	−	4	Anatomical landmark identification rate (%)	Critical landmarks characterized
15	Khan et al. (2023) [10]	Narrative review	—	Clinical	−	5	N/A (narrative)	Current advances in pituitary surgery reviewed
16	Sung et al. (2024) [39]	Meta-analysis	VR	Training	45 RCTs	1a	Pooled skill performance outcomes	High VR training effectiveness confirmed
17	Goto et al. (2023) [40]	Clinical	AR	Navigation	N = 15 patients	3	OR time (min); efficacy score (5-point scale)	Mean efficacy score 4.7/5; ~20–25% OR time reduction
18	Kawamata et al. (2002) [25]	Technical note	AR	Navigation	−	5	Navigation feasibility	First endonasal AR navigation system described
19	Yang et al. (2021) [41]	Clinical	Endoscopy	Surgery	−	4	Surgical freedom (degrees)	Approach freedom quantified across nostril configs
20	Sanker et al. (2025) [42]	Systematic review	XR	Combined	studies	1a	XR clinical and training outcomes	Extended reality applications in skull base reviewed
21	Munawar et al. (2024) [43]	Experimental	VR	Training	−	4	Task completion accuracy (%); error rate	Improved technical skills with VR immersion
22	Newall et al. (2022) [21]	Validation study	VR	Simulation	N = 15 participants (10 novice, 5 expert)	4	Face, content and construct validity scores	High fidelity VR simulator validated (mOSAT)
23	Wada et al. (2015) [44]	Clinical	CT	Anatomy	−	4	Onodi cell prevalence (%); classification	Novel sphenoid sinus classification proposed
24	Chauvet et al. (2021) [4]	Book chapter	TORS	Neurosurgery	−	5	Feasibility (narrative)	TORS role in pituitary surgery defined
25	Tortolero et al. (2025) [45]	Retrospective clinical	AR	Intraoperative	N = 18 patients	3	EOR (%); complication rate; OR time	Mean EOR 93.6%; AR visualization feasible (EndoSNAP)
26	Singh et al. (2021) [11]	Retrospective	CBCT	Anatomy	−	4	Sinus morphometric parameters (mm)	Morphometric characteristics described
27	Grunert et al. (2023) [1]	Technological	AR	Navigation	−	5	Proof-of-concept feasibility	NextLens AR navigation system described
28	Hanson et al. (2020) [2]	Clinical	—	Management	−	5	Perioperative management outcomes	Perioperative protocol for pituitary surgery described
29	Mao et al. (2024) [46]	Technological	AI + AR	Intraoperative	−	5	Real-time localization accuracy	PitSurgRT real-time AI system described
30	Fang et al. (2015) [3]	Systematic review	Endoscopy	Surgery	studies	1a	Surgical outcomes (endoscopic approach)	Purely endoscopic craniovertebral approach effective
31	Arrambide-Garza et al. (2023) [47]	Clinical	CTA	Anatomy	−	4	Safety window dimensions (mm)	Safety parameters for transsphenoidal approach quantified
32	Baker et al. (2022) [48]	Retrospective case–control	Ultrasound	Surgery	N = 27 (15 IOUS + 12 control)	3	Extent of resection (%)	IOUS improves macroadenoma resection
33	Jaworek-Troć et al. (2019) [18]	Narrative review	—	Anatomy	−	5	N/A (narrative)	Sphenoid anatomy for FESS reviewed
34	Baloiu et al. (2025) [49]	Narrative review	AI	Anatomy	−	5	N/A (narrative)	Hyperpneumatization and AI roles reviewed
35	Cho et al. (2010) [27]	Morphometric	CT	Anatomy	−	4	Pneumatization rates (%); ICA contact freq. (%)	Septa contact ICA bony shell in ~28% of cases
36	Zhang et al. (2025) [50]	Clinical	AR	Navigation	N = 5 patients (3M/2F)	3	Target registration error (mm); spatial accuracy	TRE 2.23 ± 0.57 mm; AR enhanced spatial orientation
37	Nillahoot et al. (2021) [51]	Technological	VR	Simulation	−	5	Simulator performance metrics	Novel endonasal VR simulator developed and tested
38	Rosseau et al. (2013) [26]	Educational	VR	Training	−	5	Skill acquisition metrics	Foundational VR training simulator developed
39	Campisi et al. (2023) [16]	Systematic review	AR	Navigation	studies	1a	Complication rate; navigation accuracy	AR associated with reduced intraoperative complications
40	Hudise et al. (2024) [19]	Systematic review	VR	Training	studies	1a	Training outcomes; learnability	High VR efficacy in otolaryngology surgical training
41	Novák et al. (2021) [15]	Clinical	O-arm	Navigation	N = 6 patients	3	Navigation accuracy (mm deviation)	O-arm intraoperative imaging feasible and accurate in TSS
42	Thavarajasingam et al. (2022) [52]	Systematic review	AR	Clinical	studies	1a	AR clinical outcomes vs. standard navigation	AR outcomes comparable to conventional neuronavigation
43	Santona et al. (2023) [14]	Systematic review	VR	Training	studies	1a	Simulator validity; training outcomes	VR simulators effective for transsphenoidal training
44	Bopp et al. (2022) [12]	Clinical	AR	Navigation	N = 165 patients (84 AR/81 control)	3	Intraoperative orientation accuracy	AR straightforwardly integrated; improved orientation
45	Inoue et al. (2015) [6]	Clinical	3D CT	Navigation	−	4	Anatomical identification accuracy (%)	3D CT utility in endonasal approaches confirmed
46	Saher et al. (2025) [5]	Narrative overview	VR	Training	−	5	N/A (narrative)	VR applications in neurosurgery reviewed
47	Mishra et al. (2022) [20]	Narrative overview	VR	Planning	−	5	N/A (narrative)	VR potential beyond surgical planning outlined
48	Scott et al. (2022) [22]	Narrative overview	VR	General	−	5	N/A (narrative)	VR in neurosciences—future directions
49	Heredia-Pérez et al. (2019) [24]	Experimental	VR	Robotics	−	4	Motion scaling accuracy in VR	Dynamic motion scaling in robotic TSS evaluated
50	Kim et al. (2019) [17]	Narrative overview	VR	Simulation	−	5	N/A (narrative)	VR simulators for ENT and skull base reviewed
51	Filimonov et al. (2022) [23]	Clinical	VR	Planning	N = 5 patients	4	Planning feasibility; trajectory accuracy	VR planning for craniovertebral junction in 5 cases
52	Shao et al. (2020) [13]	Educational	VR	Training	N = 30 students (15 VR/15 control)	4	Knowledge test scores; skill improvement	VR training significantly effective vs. traditional

Abbreviations: VR = virtual reality; AR = augmented reality; CT = computed tomography; CBCT = cone beam CT; CTA = CT angiography; AI = artificial intelligence; XR = extended reality; TORS = transoral robotic surgery; TSS = transsphenoidal surgery; OR = operating room. LoE = Level of Evidence (Oxford Centre for Evidence-Based Medicine): 1a = systematic review or meta-analysis; 3 = retrospective cohort or case–control study; 4 = case series, simulation study, cross-sectional, or technical study; 5 = expert opinion, narrative review, or overview article. N (sample size) not available in manuscript table; authors should verify from full text of each publication.

**Table 3:** Risk of bias and methodological quality assessment of included studies.

Author (Year)	Study Design	Assessment Tool	Key Methodological Limitations	Overall Quality Judgment
Lee et al. (2025) [28]	Clinical	NOS	Small N (NR); retrospective design; single centre; no control group; outcome assessment not blinded	**Fair (5/9)**
Bue et al. (2024) [7]	Retrospective clinical	NOS	Single centre; retrospective; no formal sample size calculation; CT assessment not blinded; potential selection bias	**Fair (6/9)**
Carl et al. (2019) [36]	Clinical cohort	NOS	Single surgeon series; retrospective subset (N = 47/288); no randomisation; fiducial vs. iCT registration not stratified; no long-term follow-up	**Fair (6/9)**
Goto et al. (2023) [40]	Prospective clinical	NOS	Small N (n = 15); no control group; self-reported efficacy scale; single centre; potential performance bias	**Fair (5/9)**
Yang et al. (2021) [41]	Clinical/cadaveric	NOS	Mixed clinical and cadaveric design; small sample; outcomes not blinded; limited generalisability	**Fair (5/9)**
Tortolero et al. (2025) [45]	Retrospective cohort	NOS	Small N (n = 18); no control group; single surgeon; retrospective; EOR assessed without blinding; no comparator arm	**Poor (4/9)**
Baker et al. (2022) [48]	Retrospective case-ctrl	NOS	Small N (n = 27); single centre; historical control group; potential selection bias; no blinding of outcome assessors	**Fair (5/9)**
Zhang et al. (2025) [50]	Clinical	NOS	Very small N (n = 5); no control group; proof-of-concept design; limited generalisability; single institution	**Poor (3/9)**
Novák et al. (2021) [15]	Clinical	NOS	Very small N (n = 6); no randomisation; single centre; no blinding; limited statistical power; pilot study	**Poor (3/9)**
Bopp et al. (2022) [12]	Retrospective cohort	NOS	Single surgeon; retrospective allocation to AR/non-AR; no randomisation; potential temporal bias; good sample size (n = 165)	**Fair (6/9)**
Inoue et al. (2015) [6]	Clinical	NOS	Small sample; retrospective; single centre; no formal control group; outcome measures not standardised	**Fair (5/9)**
Filimonov et al. (2022) [23]	Clinical case series	NOS	Very small N (n = 5); no control; descriptive design; limited to single anatomical subtype (CVJ); single institution	**Poor (3/9)**
Jaworek-Troć et al. (2022) [29]	Morphometric	NOS	Retrospective CT; bilateral measurements; adequate sample; no clinical outcome correlation; single rater	**Fair (6/9)**
Muslu et al. (2025) [8]	Morphometric	NOS	Retrospective CT; single population (Turkish); 3D Slicer methodology transparent; no clinical correlation	**Fair (6/9)**
Skvortsova et al. (2025) [31]	Cross-sectional	NOS	Convenience sample (educators); self-reported outcomes; no objective performance measure; limited to acceptability	**Fair (5/9)**
Ahmadipour et al. (2016) [9]	Clinical	NOS	Retrospective; landmark assessment not blinded; single centre; no standardised imaging protocol	**Fair (5/9)**
Newall et al. (2022) [21]	Validation study	NOS	Multi-centre; N = 15; structured validity assessment (mOSAT); expert/novice stratification; limited to simulation setting	**Good (7/9)**
Wada et al. (2015) [44]	Morphometric/clinical	NOS	Retrospective; single centre; no inter-rater reliability reported; CT-based classification lacks prospective validation	**Fair (6/9)**
Singh et al. (2021) [11]	Retrospective CBCT	NOS	CBCT-based; adequate sample; single centre; no clinical outcome correlation; population-specific results	**Fair (6/9)**
Arrambide-Garza et al. (2023) [47]	CTA morphometric	NOS	Retrospective CTA; no clinical outcome correlation; single centre; safety window not validated prospectively	**Fair (5/9)**
Cho et al. (2010) [27]	Morphometric	NOS	Retrospective; outside primary search period; no blinding; single centre; historical data; adequate N	**Fair (6/9)**
Shao et al. (2020) [13]	Educational RCT	RoB 2.0	Blinding of participants not feasible; outcome assessors not blinded; small N (n = 30); limited to students; single institution	**Some concerns**
Kenig et al. (2024) [33]	Systematic review	AMSTAR 2	Broad scope (AI in surgery generally); limited PICO specificity; heterogeneous primary studies; risk of bias not formally graded	**Moderate**
Meola et al. (2017) [35]	Systematic review	AMSTAR 2	Early publication; no protocol registration reported; limited database coverage; heterogeneous included studies; no meta-analysis	**Low**
Begagić et al. (2024) [37]	Systematic review	AMSTAR 2	PRISMA-compliant; dual screening; 19 included studies; risk of bias discussed narratively; no registration reported	**Moderate**
Sung et al. (2024) [39]	Meta-analysis	AMSTAR 2	45 RCTs included; RoB 2.0 applied; comprehensive search; broad scope (healthcare education); not transsphenoidal-specific	**Moderate–High**
Sanker et al. (2025) [42]	Systematic review	AMSTAR 2	PRISMA-compliant; recent publication; heterogeneous XR technologies; no meta-analysis; no protocol registration reported	**Moderate**
Fang et al. (2015) [3]	Systematic review	AMSTAR 2	Older search; limited databases; no quality grading of included studies; heterogeneous designs; no protocol registration	**Low**
Campisi et al. (2023) [16]	Systematic review	AMSTAR 2	PRISMA-compliant; transsphenoidal-specific; risk of bias discussed; no registration; heterogeneous platforms	**Moderate**
Hudise et al. (2024) [19]	Systematic review	AMSTAR 2	Otolaryngology-focused; PRISMA-compliant; dual screening; heterogeneous training outcomes; no protocol registration	**Moderate**
Thavarajasingam et al. (2022) [52]	Systematic review	AMSTAR 2	Transsphenoidal-specific; dual screening; heterogeneous AR systems; risk of bias narrative only; no registration reported	**Moderate**
Santona et al. (2023) [14]	Systematic review	AMSTAR 2	Training-focused; comprehensive search; PRISMA-compliant; heterogeneous simulators; no quantitative synthesis; no registration	**Moderate**
Wu et al. (2015) [30]	Technical note	Descriptive	Proof-of-concept design; small N; no clinical validation; single centre; methodology not reproducible	**Low quality**
Park & Hwang (2021) [32]	Narrative overview	Descriptive	No systematic search; narrative; no quality assessment of cited studies; expert opinion level	**Low quality**
Higa et al. (2025) [34]	Qualitative design study	Descriptive	Small N (n ≈ 11); qualitative phases; no clinical outcomes; context-specific AR prototype	**Moderate quality**
Enkaoua et al. (2025) [38]	Technological	Descriptive	Proof-of-concept; no clinical series; registration accuracy reported in phantom; external validity limited	**Low quality**
Khan et al. (2023) [10]	Narrative review	Descriptive	Narrative; no systematic search; no quality appraisal; expert opinion	**Low quality**
Kawamata et al. (2002) [25]	Technical note	Descriptive	Historical; outside search window; no formal evaluation; small N; no control	**Low quality**
Munawar et al. (2024) [43]	Experimental sim.	Descriptive	Small participant group; simulation only; no clinical transfer assessment; open-source system	**Moderate quality**
Chauvet et al. (2021) [4]	Book chapter	Descriptive	Secondary source; no original data; expert narrative; no methodology reported	**Low quality**
Grunert et al. (2023) [1]	Technological	Descriptive	Proof-of-concept; no clinical series; prototype stage; limited reproducibility data	**Low quality**
Hanson et al. (2020) [2]	Clinical review	Descriptive	Narrative; perioperative management focus; no systematic search; single-centre experience	**Low quality**
Mao et al. (2024) [46]	Technological	Descriptive	Technical feasibility only; video-based dataset; no prospective clinical testing; limited N	**Low quality**
Jaworek-Troć et al. (2019) [18]	Narrative review	Descriptive	Narrative; no systematic search; anatomical focus; no original data	**Low quality**
Baloiu et al. (2025) [49]	Narrative review	Descriptive	Narrative; no systematic search; no quality appraisal; AI roles discussed speculatively	**Low quality**
Nillahoot et al. (2021) [51]	Technological	Descriptive	Simulator development study; no participant validation; limited clinical relevance assessment	**Low quality**
Rosseau et al. (2013) [26]	Educational development	Descriptive	Outside search window; historical; small N; simulator development only; no clinical transfer	**Low quality**
Saher et al. (2025) [5]	Narrative overview	Descriptive	Narrative; no systematic search; no quality appraisal; broad scope	**Low quality**
Mishra et al. (2022) [20]	Narrative overview	Descriptive	Narrative; no systematic search; speculative future directions	**Low quality**
Scott et al. (2022) [22]	Narrative overview	Descriptive	Narrative; broad neurosciences scope; no systematic methodology	**Low quality**
Heredia-Pérez et al. (2019) [24]	Experimental sim.	Descriptive	Robotic simulation; small N; no clinical transfer; dynamic scaling evaluated in VR only	**Low quality**
Kim et al. (2019) [17]	Narrative overview	Descriptive	Narrative; no systematic search; ENT/skull base perspective; expert opinion	**Low quality**
Pušnik L et al. (2026) [53]	Retrospective	NOS	Retrospective CTA dataset; simulation only—no prospective clinical validation; bilateral measurements introduce within-subject dependency; paediatric-specific population limits generalisability; no clinical outcome correlation (safety assessed in silico only)	**Fair (6/9)**
**Good/Low risk/High confidence**	**Fair/Some concerns/Moderate**	**Poor/High risk/Low confidence**	**N/A—Technical/Narrative**	

Assessment tools: NOS = Newcastle–Ottawa Scale (0–9 stars; ≥7 = Good, 5–6 = Fair, <5 = Poor); RoB 2.0 = Cochrane Risk of Bias Tool 2.0 (domains: randomization, deviations, missing data, outcome measurement, reporting; overall: Low/Some concerns/High); AMSTAR 2 = A Measurement Tool to Assess Systematic Reviews, version 2 (critical domains: PICO, protocol, search, screening, excluded studies, risk of bias, meta-analysis; overall: High/Moderate/Low/Critically low); Descriptive = qualitative methodological appraisal applied to technical notes, narrative reviews, book chapters and educational development studies for which formal scoring tools are not applicable. Published outside the primary 2015–2025 search window; included as historical contextual reference only. Note: Quality assessments presented here are based on study design characteristics and available methodological reporting. Authors are advised to verify individual domain scores against the full text of each publication prior to submission.

## Data Availability

The original contributions presented in this study are included in the article/Supplementary Material. Further inquiries can be directed to the corresponding author.

## Supplementary Materials

The following supporting information can be downloaded at: https://www.mdpi.com/article/10.3390/jcm15114142/s1, PRISMA 2020 Main Checklist.

## Abbreviations

The following abbreviations are used in this manuscript:

VR	virtual reality
AR	augmented reality
CT	computed tomography
MRI	magnetic resonance imaging
PRISMA	Preferred Reporting Items for Systematic Reviews and Meta-Analyses
NOS	Newcastle–Ottawa Scale
RoB	Risk of Bias Tool
AI	Augmented Learning
XR	Extended Reality
TORS	Transoral robotic surgery
CBCT	Cone Beam Computed Tomography
CTA	Computed Tomography Angiography
FESS	Functional Endoscopic Sinus Surgery
ICA	Internal carotid artery
O-arm	Intraoperative 3D Imaging System
